# Peer Review of “Automating Individualized Notification of Drug Recalls to Patients: Complex Challenges and Qualitative Evaluation”

**DOI:** 10.2196/82612

**Published:** 2026-01-13

**Authors:** Robert Carter Marshall

**Affiliations:** 1 Madigan Army Medical Center, 2001 28th Street Court NW, Gig Harbor, WA, United States, 1 253-851-7945

**Keywords:** notification system, drug recalls, patient safety, medication, electronic health records, prescriptions, decision support

*This is a peer review report for “Automating Individualized Notification of Drug Recalls to Patients: Complex Challenges and Qualitative Evaluation.”*

## Round 1 Review

### General Comments

This paper [1] describes a qualitative study that aims to leverage the US Food and Drug Administration (FDA)’s Healthy Citizen prototype platform, which provides information about recalls, to automatically notify patients of relevant recalls.

### Specific Comments

#### Major Comments

1. Because of the setup of this document, it is challenging to add comments or do any editing. Not sure what happened, but it treated every line as a single object when opened in Microsoft Word. Please check your formatting.
2. On page 2, within the abstract, under Background, there is an error in the formatting. There should be a section that begins with Aim. Instead, that section is folded into the Background section and needs to be corrected.
3. On page 8, with the MyChart message, I can see why patients felt too much wording was in this layout. Surprisingly, the Patient Advisory Council agreed to this layout and the wordiness. The focus must be on the patient’s needs, not what the FDA requires. We all have seen the Prescribers' Digital Reference, and we know that the information is too dense and too small. This is similar to that in terms of format. Enlarge the font, eliminate extraneous information, and only include information that is important to the patient and in simple English. This should be pretty feasible in the formatting of the Health Citizen and/or the MyChart message.
4. You identified problems and that patients would feel obligated to contact their provider regarding the recall. Instead of exploring how to address this so that patients wouldn’t do that, thereby increasing the significant workload on the provider’s health care team, you simply gave up. I think you could have done much more with this than say, “oh, it can’t be done.” How could you word the MyChart to direct the patients only to the pharmacy that dispenses their medication instead of the primary care provider? If you didn’t ask that question, you should have. This is not the time to give up. It’s time to inquire more to find the right answers so that this could move forward and better serve both the patients and their providers.
5. It is certainly possible, given the technical requirements to create this capability, that you ran out of time and money. However, you can still benefit your team and others by focusing on the lessons learned and how you would go forward with another study.
6. One of the things that you did not do is a first round of qualitative testing and using that feedback to make changes and do a second round. Per Nielsen [2], you only need about 5 test subjects per round to get the desired, usable results. What was preventing you from doing that? Put that in the manuscript as a limitation in your Discussion.
7. Also, on page 11, in the last paragraph of the page under Discussion, there is a comment regarding patients expecting their providers to know when a recall has occurred; I think we all know this is an unreasonable expectation. Part of the communication with the MyChart message is to inform the patients not to call their provider but to call the pharmacy that dispenses their medication, which should be right on the bottle. Again, one component of the MyChart portal messaging system, as well as any other portal messaging system, is to keep patients informed and educate them. That should be a focus of this project, just as much as the technical components.
8. On page 12, in the last full paragraph on the page, you make a statement regarding the project that a strong case can be made for requiring each pill bottle to include the lot number (maybe) and National Drug Code of the pills. Since the FDA was a component of this project, that should probably have been something you recommended for the FDA to require and not leave to the state boards of pharmacy, as then you would get a patchwork of regulations. This would require the FDA to say that lot numbers and National Drug Codes are required on the bottles of all medications with an appropriate implementation period to allow for appropriate software and hardware adjustments. That is just as valuable a recommendation out of the study as any other.

## Round 2 Review

### General Comments

This paper describes a small qualitative study that aims to leverage the FDA’s Healthy Citizen prototype platform, which provides information about recalls, to notify patients of relevant recalls automatically. The project team deemed the goal unattainable and provided limited lessons learned and recommendations for potential advocacy/future solutions.

### Specific Comments

#### Major Comments

1. On page 11, in the section/paragraph beginning with “Major thematic findings included...”: these are some of the lessons learned that I mentioned in my feedback.
2. On page 12, in the paragraph beginning with “The project team concluded that...”: The “project team” felt this. Did the Patient Advisory Council and the test subjects share the same feeling?
3. On page 13, in the second paragraph on the page, in the sentence beginning with “Note that the FDA does not...”: this would clearly be a lesson learned and could be advocated for via Congress and the Department of Health and Human Services.
4. On page 13, in the second paragraph, the next sentence, beginning with “The manufacturer and lot number of dispensed medications...”: agreed. See previous comment.

## Abbreviations

FDA: Food and Drug Administration
